# Current Knowledge of Endolysosomal and Autophagy Defects in Hereditary Spastic Paraplegia

**DOI:** 10.3390/cells10071678

**Published:** 2021-07-02

**Authors:** Liriopé Toupenet Marchesi, Marion Leblanc, Giovanni Stevanin

**Affiliations:** 1 Institut du Cerveau—Paris Brain Institute—ICM, INSERM, CNRS, APHP, Sorbonne Université, Pitié-Salpêtrière Hospital, 75013 Paris, France; liriope.toupenet@icm-institute.org (L.T.M.); marion.leblanc@icm-institute.org (M.L.); 2 Neurogenetics Team, EPHE, Paris Sciences Lettres Research University, 75000 Paris, France

**Keywords:** spastic paraplegia, lysosomes, autophagy, endosomes

## Abstract

Hereditary spastic paraplegia (HSP) refers to a group of neurological disorders involving the degeneration of motor neurons. Due to their clinical and genetic heterogeneity, finding common effective therapeutics is difficult. Therefore, a better understanding of the common pathological mechanisms is necessary. The role of several HSP genes/proteins is linked to the endolysosomal and autophagic pathways, suggesting a functional convergence. Furthermore, impairment of these pathways is particularly interesting since it has been linked to other neurodegenerative diseases, which would suggest that the nervous system is particularly sensitive to the disruption of the endolysosomal and autophagic systems. In this review, we will summarize the involvement of HSP proteins in the endolysosomal and autophagic pathways in order to clarify their functioning and decipher some of the pathological mechanisms leading to HSP.

## 1. Introduction

The endolysosomal pathway is an important cellular process that has been highly conserved during evolution. It allows the internalization of cargoes and their delivery to their intended destination, including all intracellular compartments, the plasma membrane and the extracellular compartment [1]. The first step of the endolysosomal pathway is endocytosis, which allows for the internalization of macromolecules, receptors and surface proteins [2]. Once internalized from the plasma membrane, the newly formed vesicles fuse with the early endosomes (EEs). Within the EEs, various cargoes’ fates can be decided. The cargoes can be directly recycled back to the plasma membrane, sent to the trans-Golgi network (TGN) or delivered to lysosomes for degradation [1,3,4]. Therefore, the endolysosomal pathway controls the localization and levels of hundreds of proteins in the cell. Consequently, this pathway is essential for regulating major cellular functions, such as cell signaling, establishment and maintenance of cell polarity, and cell migration.

It has been shown that the endolysosomal system is interconnected with another important cellular process, namely autophagy [5]. Autophagy is a key cellular process providing vital nutrients to the cell in response to various forms of stress, such as starvation, amino acid depletion and metabolic stress. This process is also involved in the degradation of defective cellular components, such as misfolded proteins and defective organelles, representing a potential danger for the cell. To answer the variety of inducing signals, different types of autophagy can take place, including macroautophagy, microautophagy and chaperone-mediated autophagy [6,7]. Macroautophagy can be described as the sequestration of cytoplasmic contents into a double-membrane vesicle called autophagosome and the degradation of the cytoplasmic content by fusion of the autophagosome with lysosomes to form an autolysosome [8]. In the context of this review, we decided to focus mainly on macroautophagy, which we will hereafter refer to as autophagy.

Due to their importance in many cellular functions, alterations of both endolysosomal and autophagic pathways have been associated with diseases, especially neurodegenerative conditions [7,9]. In particular, we will focus on hereditary spastic paraplegia (HSP), a family of motor neuron diseases in which numerous causative genes have been linked to these pathways. We believe these diseases are good illustrations of the difficulty to establish phenotype–genotype correlations: how altering these pathways at different steps can lead to similar consequences, while sometimes the alteration of a single protein can lead to different neurodegenerative diseases.

**Figure 1:**
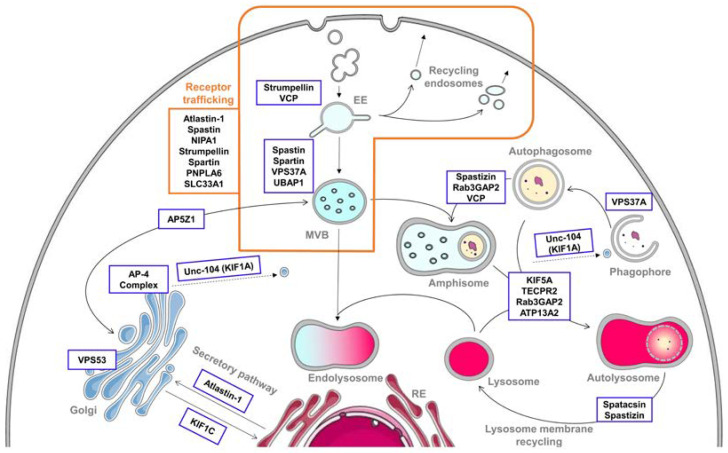
Implication of hereditary spastic paraplegia (HSP) proteins in endolysosomal and autophagic pathways. The endolysosomal pathway begins with endocytosis, consisting in the uptake of extracellular contents into vesicles. They fuse with early endosomes (EEs), which mature into multivesicular bodies (MVBs). During this fusion and maturation, the cargoes are sorted and can have three different fates. They can be directly recycled back to the plasma membrane via recycling endosomes, sent to the trans-Golgi network (TGN) or delivered to lysosomes for degradation. This process is notably highly involved in receptor trafficking, where numerous HSP proteins are implicated. For the degradation, the MVB has to fuse with the lysosome to form an endolysosome. However, MVBs can also fuse with autophagic compartments for their maturation, forming an amphisome. This hybrid structure will merge with a lysosome to give an autolysosome, where the degradation will take place, highlighting the importance of interconnections between endocytosis and autophagy. Autophagy involves the sequestration of cytoplasmic contents into a double-membrane vesicle called autophagosome and their degradation by fusion of the autophagosome with lysosomes to form an autolysosome. Once the degradation is effective, new lysosomes have to be formed using the lysosomal membrane components present in the autolysosome by the lysosome membrane recycling. All these compartments are highly interconnected, especially the Golgi and endoplasmic reticulum (ER), which also communicate with each other along the secretory pathway. Numerous HSP proteins, framed on the figure, are involved in various steps of the endocytic and autophagic pathways. Their precise involvement is summarized in Table 1. Of note, some elements of the figure are adapted from Servier Medical Art by Servier licensed under a Creative Commons Attribution 3.0 Unported License.

HSPs constitute a large family of clinically and genetically heterogeneous neurological disorders [10,11,12]. Patients present with the core clinical features of lower-limb spasticity and weakness due to degeneration in the corticospinal tracts. Clinically, there are “pure” and “complex” forms of HSPs, depending on the absence or presence of other neurological signs. Genetically, more than 70 causative genes have been described up to now, most of which are related to a relatively small number of pathological processes [11], among common genetic networks [13]. The endolysosomal and autophagic pathways are overrepresented with 24 genes whose mutations are directly involved in HSP (Table 1 and Figure 1). Multiple steps of those pathways are impaired in these disorders, not only directly by HSP protein loss of function but also as secondary consequences of the alteration of other systems. In this review, we will summarize current knowledge of the direct involvement of HSP proteins in the endolysosomal and autophagic pathways. A better understanding of the impaired cellular functions leading to HSP could help to decipher their functional organization and regulation and could also open opportunities for common therapeutic options for the various HSPs.

## 2. Alteration of the Endolysosomal Pathway in HSP

### 2.1. Endocytosis and Endosomes’ Dynamic

Endocytosis is crucial for uptake of nutrients and cell surface receptors, and for several other cellular processes [3]. Endocytosis is initiated with the invagination of the plasma membrane, leading to the formation of vesicles containing cargoes. There is diversity in the type of endocytic machinery, which classically differentiates the so-called “clathrin-dependent” or “clathrin-independent” pathways according to the requirement for the coat protein, clathrin [2].

**Figure 2:**
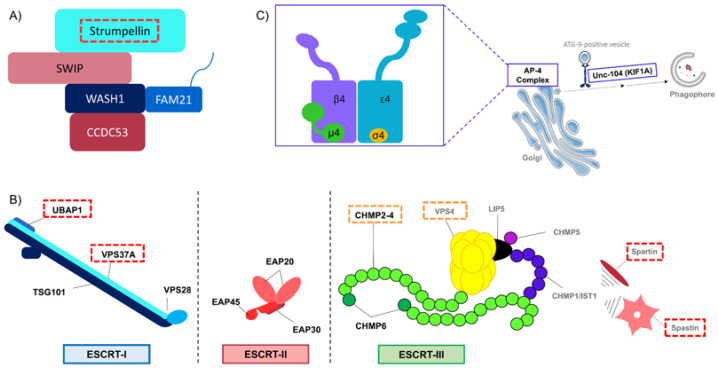
Schematic representations of the WASH, ESCRT-I to –III and AP-4 complexes. (**A**) Schematic representation of the WASH complex. The WASH complex is composed of five subunits: strumpellin (or WASHC5), SWIP, WASH1, CCDC53 and FAM21. The WASH complex plays a critical role in endosome tubulation and fission. Mutations in the WASHC5/strumpellin subunit (highlighted by a dashed red frame) led to a pure form of HSP, SPG8. (**B**) Schematic representation of the ESCRT system from ESCRT-I to ESCRT-III. The ESCRT system plays a central role in intracellular trafficking. Notably, the ESCRT machinery is involved in the formation of MVBs since they are implicated in the sorting of cargoes and the formation of ILVs. ESCRT-I is a tetrameric complex composed of four subunits with two core subunits: TSG101 and VPS28 and of two additional subunits: MVB12A/B, UBAP1, UBA1L or UMAD1 and one isoform of VPS37 (A/B/C/D). ESCRT-II is also a tetramer composed of EAP45, EAP30 and two EAP20. Finally, ESCRT-III is composed of four main proteins, CHMP2A/B, CHMP3, CHMP4A/B/C, and CHMP6. To those proteins, can be associated at least three ESCRT-III related proteins: CHMP1A/B, CHMP5 and IST1. On the right panel are also represented VPS4 and its cofactor LIP5 as well as spastin and spartin which are interactors of ESCRT-III and ESCRT-III related proteins. Importantly, VPS37A, UBAP1, spastin and spartin are mutated in HSP, corresponding to SPG53, SPG80, SPG4 and SPG20 respectively. Of note, mutations in the gene encoding for CHMP2B can give rise to other neurodegenerative conditions: amyotrophic lateral sclerosis (ALS), frontotemporal dementia (FTD) and ALS-FTD. In addition, mutations in one isoform of VPS4, VPS4A, has been found in a multisystemic disease with abnormal neurodevelopment. The proteins mutated in HSP or other neurological disorders are highlighted by dashed red or orange frames, respectively. (**C**) Schematic representation of the AP-4 complex and ATG-9-positive vesicles transport. The AP-4 complex specifically localizes to the TGN where it would allow the sorting of transmembrane proteins into cargoes and the recruitment of their transport machineries for their delivery to their destined localization. Among those proteins can be found ATG9A in mammals that will be delivered to the phagophore in ATG9A-positive vesicles. In the zoom box, there is a representative schematic model of AP-4 with the four subunits composing the complex: ε4, β4, μ4 and σ4, respectively encoded by *AP4E1*, *AP4B1*, *AP4M1* and *AP4S1*. Mutations in these four genes lead to severe neurodevelopmental forms of HSP (SPG47, SPG50, SPG51 and SPG52, respectively). In C. elegans, the motor protein Unc-104, the homologue of KIF1A, mutated in SPG30, would participate in the transport of ATG-9-positive vesicles to the phagophore for the autophagosome biogenesis. Of note, some elements of the figures are adapted from Servier Medical Art by Servier licensed under a Creative Commons Attribution 3.0 Unported License.

One of the clathrin-independent pathways is the caveolae and caveolin1 (CAV1) dependent pathway. CAV1 is enriched in caveolae, small invaginations of the plasma membrane that have various cellular functions, particularly lipid regulation via the ganglioside GM1 [2,14]. Of note, ganglioside dynamics are affected indirectly in SPG11 (for Spastic Paraplegia Gene 11), a frequent autosomal recessive subtype of HSP [15], through accumulation of GM2 species in lysosomes [16], and directly in SPG26, another HSP form, because of the loss of function of one key enzyme of ganglioside metabolism, B4GALNT1 [17]. More interestingly, the CAV1-dependent pathway is affected by mutations in the *SPG8* gene, which cause a pure form of spastic paraplegia [18]. *SPG8* encodes for the strumpellin protein, also called WASHC5, a member of the hetero-pentameric Wiskott–Aldrich syndrome protein and SCAR homolog (WASH) complex. WASH is composed of five subunits: WASH1, strumpellin, FAM21, KIAA1033(SWIP) and CCDC53 (Figure 2A) [19,20]. WASH activates the Arp2/3 complex on the surface of endosomes. Arp2/3 organizes the actin network and is required for endosome tubulation and fission [21]. WASH is involved in different steps of the endosomal tubulation and sorting dynamics. Thus, WASH depletion can lead to a wide variety of phenotypes, ranging from exaggerated tubulation to abolishment of tubulation associated with endosome enlargement [22]. The SPG8 mutations do not affect strumpellin interaction with the WASH complex or the WASH complex localization in the cell. However, in cells depleted in strumpellin or with mutated strumpellin, endosomal tubulation is impaired and an abnormal lysosomal morphology can be observed [23,24,25]. Moreover, strumpellin interacts with CAV1 and is necessary for maintenance of CAV1 abundance. Strumpellin-depleted cells have reduced levels of CAV1 due to an increase in its degradation. Indeed, CAV1 is ubiquitinated and targeted to lysosomes for degradation, but when protein degradation at lysosomes is inhibited, CAV1 abundance is restored in strumpellin-depleted cells. Thus, maintenance of the CAV1 protein level depends on strumpellin activity. The perturbation of caveola endocytosis in the case of strumpellin deficiency is certainly a primary event of SPG8 pathogenesis [25].

**Table 1:** HSP proteins involved in the endolysosomal system and autophagy. The cellular consequences mentioned here are only in the context of this review, and therefore only include the function of the protein in the endolysosomal and autophagic pathways. All the references to the SPG numbers and their clinical involvement can be found in Hedera et al. or Boutry et al. [11,12].

SPG	Gene	Protein	Relevant Section in the Text	Contribution in the Pathway	Cellular Consequences of Mutation/Depletion	References
SPG3A	*ATL1*	Atlastin-1	2.2 Receptor trafficking 2.3 Secretory pathway	Regulation of BMP signaling ER-Golgi trafficking	Overactivation of BMP signaling Impairment of ER-Golgi trafficking and Golgi morphogenesis	[26,27,28,29]
SPG4	*SPAST*	Spastin	2.1 Endocytosis and endosomes’ dynamic 2.2 Receptor trafficking	Regulation of ESCRT-III	Perturbation of endosomal tubulation Overactivation of BMP signaling	[30,31,32,33,34]
SPG6	*NIPA1*	NIPA1	2.2 Receptor trafficking	Regulation of BMP signaling	Overactivation of BMP signaling	[28,35,36]
SPG8	*KIAA0196/ WASHC5*	Strumpellin/ WASHC5	2.1 Endocytosis and endosomes’ dynamic 2.2 Receptor trafficking	Member of WASH complex	Perturbation of clathrin-independent pathway Impairment of endosomal tubulation	[23,24,25]
SPG10	*KIF5*	KIF5A	3.2 Autophagosome—lysosome fusion	Kinesin, motor protein	Impairment of the axonal transport and autophagic flux	[37]
SPG11	*SPG11*	Spatacsin	3.3 Lysosome membrane recycling	Recruitment of Dynamin Interacts with spastizin and AP-5	Autophagy defects due to reduction of autolysosome tubulation Accumulation of autophagic compartments Defective lysosomal clearance of gangliosides	[16,38,39,40,41]
SPG15	*ZFYVE26*	Spastizin	3.3 Lysosome membrane recycling 3.4 Crossroads between endocytic and autophagic pathways	Interacts with spatacsin and AP-5 Interaction with Rab5A and Rab11	Autophagy defects due to reduction of autolysosome tubulation Accumulation of autophagic compartments Altered maturation of autophagosomes	[38,39,40,42,43]
SPG20	*SPART*	Spartin	2.1 Endocytosis and endosomes’ dynamic 2.2 Receptor trafficking	Regulation of ESCRT-III	Perturbation of endosomal trafficking Overactivation of BMP signaling	[44,45,46,47,48,49]
SPG30	*KIF1A*	KIF1A/Unc-104	3.1 Autophagosome biogenesis	Kinesin, motor protein	Impaired transport of ATG-9-positive vesicles leading to defects in autophagosome biogenesis	[50]
SPG39	*PNPLA6*	PNPLA6	2.2 Receptor trafficking	Regulation of BMP signaling	Overactivation of BMP signaling	[51]
SPG42	*SLC33A1*	SLC33A1	2.2 Receptor trafficking	Regulation of BMP signaling	Overactivation of BMP signaling	[52]
SPG47	*AP4B1*	AP4B1	2.3 Secretory pathway 3.1 Autophagosome biogenesis 3.3 Lysosome membrane recycling	Subunit of AP-4 complex	Impairment of ATG9A’s sorting and thus autophagosome biogenesis	[53,54,55]
SPG48	*AP5Z1*	AP5Z1	3.3 Lysosome membrane recycling 3.4 Crossroads between endocytic and autophagic pathways	AP-5 subunit spatacsin and spastizin interactor	Reduction of autolysosome tubulation Impaired endolysosomal system due to accumulation of endolysosomes Impairment of CIMPR trafficking towards TGN	[56,57,58,59]
SPG49	*TECPR2*	TECPR2	3.2 Autophagosome—lysosome fusion	Interactor of HOPS and ATG8 family members	Accumulation of autophagosomes due to impaired autophagosome—lysosome fusion	[60,61]
SPG50	*AP4M1*	AP4M1	2.3 Secretory pathway 3.1 Autophagosome biogenesis 3.3 Lysosome membrane recycling	Subunit of AP-4 complex	Impairment of ATG9A’s sorting and thus autophagosome biogenesis	[53,54,55]
SPG51	*AP4E1*	AP4E1	2.3 Secretory pathway 3.1 Autophagosome biogenesis 3.3 Lysosome membrane recycling	Subunit of AP-4 complex	Impairment of ATG9A’s sorting and thus autophagosome biogenesis	[53,54,55]
SPG52	*AP4S1*	AP4S1	2.3 Secretory pathway 3.1 Autophagosome biogenesis 3.3 Lysosome membrane recycling	Subunit of AP-4 complex	Impairment of ATG9A’s sorting and thus autophagosome biogenesis	[53,54,55]
SPG53	*VPS37A*	VPS37A	2.1 Endocytosis and endosomes’ dynamic 3.1 Autophagosome biogenesis	Subunit of ESCRT-I	Perturbation of endosomal sorting Altered capacity to recruit ESCRT-I subunits at the PAS leading to impaired autophagosome closure	[62,63]
SPG58	*KIF1C*	KIF1C	2.3 Secretory pathway	Kinesin, motor protein	Impairment of Golgi-ER transport	[64,65,66]
SPG69	*RAB3GAP2*	Rab3GAP2	3.4 Crossroads between endocytic and autophagic pathways	Subunit of Rab3GAP complex	Autophagy defects	[67,68]
SPG78	*ATP13A2/ PARK9*	ATP13A2	3.2 Autophagosome—lysosome fusion	Still unclear	Autophagy defects due to accumulation of autophagic compartments	[69,70]
SPG80	*UBAP1*	UBAP1	2.1 Endocytosis and endosomes’ dynamic	Subunit of ESCRT-I	Perturbation of endosomal sorting	[71,72]
\	*VCP*	VCP	2.1 Endocytosis and endosomes’ dynamic 3.4 Crossroads between endocytic and autophagic pathways	Interaction with strumpellin Autophagosome maturation	Perturbation of strumpellin localization and function Autophagy defects	[73,74,75,76]
\	*VPS53*	VPS53	2.3 Secretory pathway	Subunit of GARP complex	Still unclear	[77]

Interestingly, the gene encoding for the protein valosin-containing protein (VCP), an interactor of strumpellin, can cause several neurodegenerative diseases such as amyotrophic lateral sclerosis (ALS), Charcot–Marie–Tooth (CMT) disease and notably HSP, when mutated [73,74,75,78,79]. VCP is a ubiquitously expressed AAA–ATPase protein with multiple cellular functions that include vesicular trafficking and protein degradation. Although VCP function is still unclear, its interaction with strumpellin could be necessary for normal functioning of strumpellin. Mutant VCP may then alter strumpellin function and even sequester it, since aggregates of VCP and strumpellin have been found in muscles biopsies from patients with VCP mutations [75]. Moreover, VCP-positive inclusion bodies have been found in several neurodegenerative diseases, such as Parkinson’s disease, amyotrophic lateral sclerosis (ALS) and spinocerebellar ataxia type 3. Additionally, strumpellin has been identified in pathological protein aggregates in inclusion body myopathy with Paget disease and frontotemporal dementia (IBMPFD), various myofibrillar myopathies and in cortical neurons of a Huntington’s disease mouse model [75,80].

The degradation pathway starts with the formation of late endosomes from the maturation of early endosomes. Cargoes that will be degraded are contained in intraluminal vesicles (ILV). Aggregation of several ILVs leads to the formation of a late endosome also called multivesicular body (MVB). The ubiquitination of ILVs is the key signal for their degradation. Indeed, ubiquitinated cargoes are trafficked and delivered to MVBs and lysosomes for degradation, whereas non-ubiquitinated ones are sorted and transported to the TGN or recycled back to the plasma membrane. The detection of the ubiquitination, the packaging of the cargoes into ILVs and the formation of the vesicles are mediated by the multiprotein complexes belonging to the endosomal sorting complexes required for transport (ESCRT) family [1,81]: ESCRT-0, -I, -II and –III (Figure 2B) [82,83,84]. The ESCRT system has been shown to play a central role in intracellular trafficking.

We know that defects in the ESCRT system can cause various neurodegenerative diseases and HSPs are no exception. For instance, mutations in two subunits of ESCRT-I lead to HSP. ESCRT-I composition can change depending on the cellular context and can be composed of VPS37A, UBAP1, TSG101 and VPS28 [85]. Indeed, mutation in *SPG53*, encoding VPS37A, causes a complex HSP [62] and mutation in *SPG80*, encoding UBAP1, causes a pure HSP [71,72]. Variation in clinical symptoms might suggest an additional role for these proteins besides involvement in ESCRT-I, notably a function in autophagy for VPS37A (see autophagy section), or redundancy. UBAP1 interacts directly with ubiquitin on endosomes and thus participates in driving cargoes into the degradation pathway. Studies on mouse hippocampal neurons have shown that mutation in *Ubap1* alters UBAP1 recruitment to endosomes and then its function in endosomal sorting [71]. Similar results were obtained in cortical neurons derived from transgenic Ubap1 flox mice in which disruption of UBAP1 leads to dysregulation of both early-endosome processing and ubiquitinated protein sorting [72]. Moreover, in zebrafish, lack of UBAP1 leads to abnormal morphology, impaired motor neuron outgrowth and therefore decreased mobility [72]. Therefore, perturbation of the detection of cargoes fated to degradation seems deleterious for cells, and specifically for neurons, in HSP.

Unlike the other ESCRT complexes, ESCRT-III is not localized to endosomes but exists in an autoinhibited state in the cytoplasm. This complex is made up of at least four subunits, all members of the same protein family: “charged multivesicular body proteins” (CHMP). Activation of ESCRT-III occurs when the ESCRT-II subunit EAP20 (VPS25) binds to CHMP6 (VPS20), initiating ESCRT-III recruitment to the endosomes and complex formation [85]. ESCRT-III is responsible for the final sorting of cargoes and plays a direct role in the generation of luminal vesicles. ESCRT-III filaments surround the cargo, and its assembly induces the dissociation of the other ESCRT complexes. While ESCRT-III assembly stabilizes the membrane neck of a growing ILV, VPS4 binding to ESCRT-III subunits constricts the neck and facilitates its release from the membrane [86,87]. Alterations in ESCRT-III functions are known to induce neurological disorders. Indeed, mutations in *CHMP2B*, a gene encoding one of the four major proteins of ESCRT-III complex, can cause frontotemporal dementia (FTD), ALS or FTD-ALS [88,89,90,91]. In addition, mutations in the gene encoding VPS4A are found in patients with a multisystem disease with abnormal neurodevelopment [92].

Interestingly, the most common HSP genetic entity is due to mutations in *SPG4*, encoding spastin, which interacts with CHMP1B, a member of ESCRT-III [30]. The interaction occurs in a region containing an N-terminal microtubule interacting and trafficking (MIT) domain. This domain is shared with several proteins that have defined roles in membrane trafficking, including VPS4. Spastin’s MIT domain seems to be especially important since mutations in this domain lead to impaired endosomal tubule fission, altered endosome-to-Golgi Mannose-6-Phosphate Receptor (M6PR) traffic and abnormal lysosomal morphology [31,93]. In addition, spastin interacts with IST1, an ESCRT-III-like protein, and the lack of IST1 in cells also leads to an increase in endosomal tubulation. Moreover, abnormal endosomal tubulation has also been observed in spinal motor axons of zebrafish depleted of spastin or IST1. Furthermore, altering endosomal tubulation causes disturbance in receptor sorting and trafficking, such as BMP or transferrin receptors [32,33]. These results support the idea that spastin plays a role in intracellular transport and membrane trafficking.

Another HSP, caused by mutations in *SPG20*, could be associated with the ESCRT system. The encoded protein, spartin, localizes to endosomes [44,45] and also has an MIT domain, predicting a role in endosomal trafficking [46]. In addition, spartin interacts with the spastin interactor, IST1. Spartin seems to be an inhibitor of IST1 activity, possibly by preventing its interaction with spastin. Overexpression of IST1 in primary rat cortical neurons induces an increase in the number of axon branches, whereas overexpression of spartin in *Spg20*^−/−^ primary neurons significantly decreases the number of axon branches [47]. However, the mechanisms underlying ESCRT-mediated effects on axon branching still need to be deciphered. Spartin also interacts with alsin, which has an important role in motor neuron integrity and accounts, in the case of loss of function mutations, for another motor neuron disease, ALS, and more rarely for HSP phenotypes [94].

Once the molecules destined to be degraded have been sorted, the MVBs fuse with lysosomes to form endolysosomes. In this compartment, the degrading enzymes formerly present in lysosomes will therefore be able to degrade the molecules contained in MVBs, marking this step as the end of the endolysosomal degradation pathway [95].

### 2.2. Receptor Trafficking Impairment

As we have seen, alteration of endosome tubulation is a recurrent pattern in HSP and has a direct impact on the fate of cargoes. Perturbation of sorting and recycling of cargoes, notably receptors, could be a key pathological mechanism of several HSPs. Indeed, failure of endosomal tubule fission can cause defective sorting of various receptors. For example, in strumpellin-depleted cells, the subcellular distribution of β-2-adrenergic receptors is altered [24]. Furthermore, spartin interacts with endocytic trafficking protein EPS15 and plays a role in intracellular trafficking of epidermal growth factor receptors (EGFR) [48].

More interestingly, the bone morphogenic protein (BMP) signaling pathway has progressively emerged as a common pathogenic mechanism in at least six HSP subtypes: SPG6 (NIPA1), SPG3A (atlastin-1), SPG4 (spastin), SPG20 (spartin), SPG39 (PNPLA6), SPG42 (SLC33A1) [26,27,34,35,36,49,51,52,96]. BMP constitutes a group of phylogenetically conserved growth factors and belongs to the transforming growth factor beta (TGFβ) superfamily [97]. First detected in extracts of bones, the BMP signaling pathway has a critical role in the nervous system. Initially, BMPs inhibit proliferation of neural precursors and promote the first steps of neuronal differentiation. Furthermore, in post-mitotic cells, they modulate neuronal subtype specification, promote dendritic and axonal growth and induce synapse formation and stabilization [98,99].

The first link between HSP and BMP signaling was made in 2007. Indeed, loss of spichthyin (SPICT), the *Drosophila melanogaster* orthologue of NIPA1, leads to upregulation of BMP signaling. SPICT localizes with early endosomes, interacts with BMP receptors, promotes their internalization from the plasma membrane and, finally, leads to BMP signaling inhibition [35]. In mammalian cells, NIPA1 physically interacts with the type II BMP receptor (BMPRII) and promotes its endocytosis and lysosomal degradation [36]. Mutations in *ATL1,* encoding for atlastin-1,have been found as causative in a subtype of HSP, SPG3A, and in hereditary sensory and autonomic neuropathy (HSAN) [12,100]. This HSP related protein strongly interacts with NIPA1 and BMPRII [28]. Even if atlastin-1 expression is not necessary for trafficking of BMPRII, the presence of mutant forms of atlastin-1 results in a complete absence of BMPRII from the plasma membrane and its clustering in the cytoplasm, suggesting a dominant negative effect [27]. Moreover, knockdown of *Atl1* gene (coding for atlastin-1) in zebrafish induces a loss of motility of the zebrafish larvae, associated with impairment of branching axons. Overexpression and knockdown studies have shown that atlastin-1 inhibits BMP signaling. Atlastin-1 co-localizes with type I BMP receptors (BMPRI) in endosomal structures along neurites implying a role in BMP receptor trafficking regulation [26].

The HSP proteins spastin and spartin are inhibitors of BMP signaling. How they participate in the modulation of BMP signaling is not absolutely known, but both are involved in endosomal tubulation, which is associated with trafficking and recycling of receptors. A study in zebrafish depleted in spastin has shown motor circuit formation alterations and have linked a part of these defects to BMP signaling over-activation [34]. In Drosophila, spartin is required for downregulation of the BMP receptor Wit, which initiates a retrograde signal controlling microtubule stability and synaptic growth. In this model, both spartin depletion and elevated BMP signaling cause progressive neurodegeneration [49]. Finally, an upregulation of BMP signaling has also been noted in a mouse model of SPG42 (SLC33A1) [52] and in *Pnpla6* (SPG39) knockdown in zebrafish [51]. The links between these two proteins and BMP signaling regulation need further investigation.

In conclusion, all these studies show a strong link between receptor trafficking, particularly involved in BMP signaling regulation, and HSP. BMP signaling has a critical role in motor circuit architecture and stability and small alterations in this pathway can cause major effects on motor axons and finally lead to neurodegeneration more or less associated with abnormal neurodevelopment as shown in some animal models.

### 2.3. Secretory Pathway

The Golgi apparatus in eukaryotic cells is composed of flattened and fenestrated membrane disks, called cisternae. Several cisternae, between 3 and 20, are aligned in parallel to form a stack. The Golgi stack is subdivided in three compartments: *cis*, *medial* and *trans*. Two networks responsible for the main tasks of this apparatus can be identified. The *cis*-Golgi network (CGN) is situated closest to the endoplasmic reticulum (ER) and is responsible for receiving ER-derived transport vesicles and shipping ER resident proteins back to the ER. On the contrary, the cisterna in the most *trans* position is continuous with a tubular, branching and reticulating compartment termed the TGN. The TGN is involved in the final stage of sorting, packing and delivering of most secretory proteins to their destinations. Moreover, cargoes from the early and late endosomes are targeted to the TGN via the retrograde route. Therefore, the TGN has conventionally been viewed as the main cargo sorting station where proteins and lipids are sorted and targeted to various downstream destinations [4,101].

Once the endosome-derived transport carrier reaches the proximity of the TGN, the carrier-Golgi interaction is mediated by tethers, one of them being the Golgi-associated retrograde proteins (GARP) complex. GARP is a heterotetrameric-tethering complex and consists of four subunits: VPS51, VPS52, VPS53 and VPS54 [102]. Dysfunction of the GARP complex leads to defects in both retrograde trafficking and anterograde trafficking. Mutations in the gene encoding the VPS54 subunit, which lead to a drastic reduction in levels of VPS54 and disturb the GARP complex assembly, result in spinal muscular atrophy in the “wobbler” mouse that is considered as a model for ALS [103]. Recently, mutations in two others GARP complex subunits, VPS53 and VPS51, have been associated with a complex form of HSP and a neurodevelopmental disorder in patients, respectively, demonstrating the importance of this complex in the pathway [77,104].

Cytosolic cargo adaptors play a fundamental role in protein sorting at the TGN. Five adaptor proteins (APs) have been identified in eukaryotic cells, and three of them (AP-1, AP-3 and AP-4) are involved in protein sorting at the TGN [105]. Each AP complex is formed of four subunits. The AP-4 complex is composed of four subunits encoded by four different genes: *AP4E1*, *AP4B1*, *AP4M1* and *AP4S1* (Figure 2C) [50,53,54,55,106]. Mutations in the genes encoding all subunits of AP-4 lead to severe neurodevelopmental forms of HSP (SPG47, SPG50, SPG51 and SPG52, respectively) [107,108,109,110,111]. In this context, mutations of AP-4 subunits mainly lead to autophagy defects that will be detailed in the autophagy section of this review.

Proteins destined for secretion, plasma membrane or intracellular organelles are synthesized in the ER and transported along the secretory pathway through the Golgi complex by coated vesicular carriers. These vesicles fuse with the CGN. In the opposite way, this flow of membranes needs to be counterbalanced by a reverse membrane transport pathway from Golgi to ER. This involves vesicles traveling along microtubules. Interestingly, mutations in proteins involved in Golgi-ER (or ER-Golgi) traffic can lead to HSP. Indeed, mutations in the gene encoding atlastin-1 (*SPG3A*), another HSP protein localized with *cis*-Golgi and ER markers, affect trafficking at the ER/Golgi interface and lead to impaired Golgi morphogenesis [29]. Similarly, mutations in a gene of the kinesin family, *KIF1C*, cause a complex HSP (SPG58) [64,65]. Little is known about the precise functions of the KIF1C protein but it is associated with the Golgi membranes and required for the retrograde transport of Golgi vesicles to the ER [66].

Finally, the ER is a major organelle involved in numerous cellular functions and is widespread in cells through a network of interconnected sheets and tubules. This network allows the ER to communicate with the other organelles and contribute to their functions, notably to obtain effective endocytic and endolysosomal pathways [112]. Among HSP proteins, many have been directly implicated in ER dysfunctions. In particular, the most represented ones are: atlastin-1 (SPG3A), reticulon-2 (SPG12), REEP1 (SPG31), REEP2 (SPG72) and erlin-2 (SPG18/37). Their precise involvement in those processes will not be dealt with here since it has already been extensively described recently [11,113,114,115].

## 3. Autophagy Defects in HSP

### 3.1. Autophagosome Biogenesis

Upon autophagy induction, Autophagy-related (ATG) proteins are recruited to the location of the defective material, called the phagophore assembly site (PAS), to initiate the formation of the phagophore. The phagophore can be described as a double-membrane saccule composed of isolation membranes budding from the ER and other sources of membranes [7,116]. The initiation step of phagophore formation is followed by a nucleation step and an elongation step, leading to the formation of a fully closed autophagosome sequestering the intracellular material [117]. Many ATG proteins are implicated in the initiation, nucleation and elongation of the phagophore. Notably, the arrival of ATG9-containing vesicles at any of these steps promotes the input of additional membrane material (lipids, proteins) to form the autophagosome [116].

ATG9, ATG9A in mammals, is a transmembrane protein belonging to the core machinery of autophagosome formation. When autophagy is not induced, this protein mainly localizes to the TGN and partly to the endosomes. However, upon autophagy induction, ATG9A is delivered to the PAS, by the intermediary of vesicles, to regulate the elongation of the phagophore [118]. As shown above, each of the four subunits of AP-4 complex has been involved in a complex form of HSP. The AP-4 complex is specific to the TGN, where it would allow the sorting of transmembrane proteins into cargoes and the recruitment of their transport machineries for their delivery to their destined localization [53,54]. In various cellular types, the depletion of each of the AP-4 subunits leads to the mislocalization of ATG9A with its retention at the TGN and its depletion in the periphery [53,54]. AP-4-dependent sorting and localization of ATG9A would be especially important in neuronal cells since an impairment in basal and stress-induced autophagy has been observed in iPSC-derived cortical neurons from SPG47 patients but not in fibroblasts derived from patients mutated in AP-4 subunits [53]. Consistently, a reduction of the axonal autophagosome formation with ER accumulation in axonal swellings has been observed in neurons of the *Ap4e1*^−/−^ mouse model [55]. Of note, AP-4 is not only involved in ATG9A sorting but takes charge of other cargoes, such as the AMPA-type glutamate receptor, which has been reported with impaired trafficking in the *Ap4b1*^−/−^ mouse model. However, the fact that the phenotype of the *Ap4e1*^−/−^ mouse model is similar to the *Atg9*^−/−^ mouse model, even if less severe, suggests an important role of ATG9A trafficking in AP-4-linked HSP [54]. This is also supported by the potential involvement of another HSP protein, KIF1A, in ATG9A trafficking. ATG9A-positive vesicles must be transported to their sites of action and especially to the axon terminals where ATG9A is highly localized [119]. It has been shown that in C. elegans, Unc-104, the orthologue of the mammalian KIF1A member of the Kinesin-3 family, is involved in the transport of ATG-9 to neurites [50]. Pathological variants in the gene encoding for KIF1A are involved in the development of autosomal recessive and dominant spastic paraplegia, namely SGP30 [120,121]. Of note, mutations in this gene can also give rise to a subtype of HSAN [100]. In vertebrates, ATG9A has not yet been identified as a cargo of KIF1A but could be an interesting candidate [122].

After its formation and elongation, the phagophore has to be closed to form the autophagosome but this process is still relatively unknown. However, it was recently shown that components of the ESCRT-I and ESCRT-III machineries, implicated in endosome tubulation as seen before, are involved in autophagosome closure [63]. Among the ESCRT proteins required, VPS37A (SPG53) would be involved in the promotion of autophagosome closure by recruiting the ESCRT-III subunit CHMP2A and the AAA ATPase VPS4 [63].

### 3.2. Autophagosome–Lysosome Fusion

Once the autophagosome has formed, a maturation step takes place. To be able to fuse, the autophagosome and lysosomes have to encounter one another in the cell despite their generally different cellular positions [123]. Bi-directional transport of both organelles then has to take place. Autophagosomes and lysosomes are transported by a kinesin-dependent plus-end transport, notably the kinesin-1/KIF5 [123,124]. Three isoforms of KIF5 exist: KIF5A, KIF5B and KIF5C [125]. The ubiquitously expressed subtype KIF5B has been shown to be largely involved in autophagy and autophagic lysosome reformation (ALR) [126,127]. KIF5A is a neuronal subtype of KIF5 that is involved in various allelic diseases when a mutation occurs in its corresponding gene: a form of HSP, SPG10, as well as ALS and CMT disease [125,128,129,130,131]. KIF5A has been mostly implicated in axonal transport of neurofilaments, mitochondria and amyloid precursor protein [132,133,134,135]. In addition to those roles, a recent study demonstrated that KIF5A is reduced in Neuro-2a cells treated with a neurotoxicity-inducing drug. That deficit would lead to an impairment of the autophagic flux due to a KIF5A-dependent axonal transport deficiency, suggesting that KIF5A could be important in autophagy [37].

The proteins of the ATG8 family, notably composed of the LC3 (light chain 3) proteins, are necessary to recruit interactors to initiate the transport, tethering and fusion of the autophagosome with lysosomes [123,136]. Among the necessary actors can be found the homotypic fusion and vacuole protein sorting (HOPS) tethering complex which is composed of six subunits: VPS39, VPS11, VPS18, VPS16, VPS33 and VPS41. This complex allows the Soluble N-ethylmaleimide-sensitive factor attachment protein receptors (SNARE)-mediated fusion between the outer membrane of the autophagosome and the lysosomal membrane. Notably, the syntaxin 17 (STX17) and synaptosomal-associated protein 29 (SNAP29) autophagosomal SNAREs and the lysosomal vesicle-associated membrane protein 8 (VAMP8) are involved in this process [60,116,137]. Mutations in three subunits of the HOPS complex, VPS11, VPS16 and VPS41, have been linked to neurological disorders. For example, patients mutated in *VPS11* present with signs of hypomyelination that can be associated with leukoencephalopathy [138,139,140,141]. Mutations in *VPS11* could also give rise to dystonia as observed in patients mutated in *VPS16* and *VPS41*, associated with ataxia for the latter [141,142,143,144,145,146]. Of note, mutations in SNAP29 are also involved in neurological disorders since they can lead to the development of the cerebral dysgenesis, neuropathy, ichthyosis, and keratoderma (CEDNIK) syndrome [147,148,149].

The tectonin beta-propeller repeat containing 2 (TECPR2) protein has been identified as an interactor of the ATG8 family and of the HOPS complex [150]. Mutations in the gene coding for TECPR2 have been identified in a form of HSP, SPG49, and have been proposed as involved in a subtype of HSAN [12,151]. A link between *SPG49* mutations and autophagy has been made by the observation of a defect in autophagy in SPG49 patient-derived fibroblasts [152]. Recently, it has been proposed that TECPR2 regulates autophagosome–lysosome tethering and fusion by binding the autophagosome with its LC3-interacting region (LIR) and its interaction with HOPS [60]. The precise molecular function of TECPR2 in this model still has to be explored but it is consistent with the observation of autophagosome accumulation in the brain and spinal cord of the *Tecpr2*^−/−^ mouse model, suggesting an altered delivery of autophagosomes to lysosomes [61]. Interestingly, neurons of the *Tecpr2*^−/−^ mouse model display age-dependent axonal spheroids or swellings with accumulation of autophagic material [61].

Another HSP protein could be involved in this process. ATP13A2 is a lysosomal transmembrane P5B-type ATPase localized at acidic compartments, which is notably involved in SPG78 and Parkinson’s disease [69,153,154,155]. Fibroblasts from patients carrying mutations in *ATP13A2* present signs of autophagic pathway defects with an accumulation of lysosomal structures and abnormal lysosomal storage material [69]. The precise molecular involvement of ATP13A2 in autophagy is still unclear but a recent study in mouse embryonic fibroblasts (MEFs) of *Atp13a2*^−/−^ mouse and ATP13A2-depleted HeLa cells suggests that it is required for autophagosome–lysosome fusion. In these cells, ATP13A2 depletion leads to both autophagosome and lysosome accumulation, indicating an autophagy defect that would notably lead to a defect in clearance of damaged mitochondria [70].

### 3.3. Lysosome Membrane Recycling

After formation of the autolysosome, the hydrolases and acidic environment provided by the lysosome will allow the degradation of the internal membrane and of the autophagosomal cargoes [116]. Following this fusion, the final step of the autophagic process is the recycling of some lysosomal components to allow the formation of a new pool of lysosomes, either by vesiculation in conditions of basal autophagy [38,156] or by tubulation in ALR in conditions of stress-induced autophagy [157,158]. Several HSP proteins have been implicated at different levels in the lysosomal reformation, notably spatacsin (SPG11), spastizin (SPG15), AP5Z1 (SPG48) and the AP-4 complex [38,56,57,159].

Spatacsin and spastizin are frequently mutated proteins in autosomal recessive forms of HSP. Spatacsin is encoded by *SPG11* and mutated in three allelic motor neuron diseases: SPG11, ALS and CMT disease [15,79,160,161,162]. Spastizin is encoded by *ZFYVE26* and mutated in SPG15 [163]. At the protein level, SPG11 and SPG15 interact with each other, might have an interdependent expression and stability [39] and both have also been shown to stably interact with the adaptor protein complex 5 (AP-5) [164]. The molecular functions and localizations of spatacsin and spastizin are still debated [165,166,167,168]. Among the propositions, the localization of those proteins at the late endosome/lysosomal compartments is especially interesting and is supported by the fact that spastizin possesses a FYVE domain able to target proteins to intracellular membranes enriched for phosphatidylinositol 3-phosphate, such as endosomal membranes [169]. Both spatacsin and spastizin have notably been involved in ALR since their mutations lead to a phenotype associated with ALR defects [38]. In conditions of stress-induced autophagy, ALR starts by the formation of tubules containing lysosomal membrane components, which are extruded from the autolysosome. From those tubules will bud vesicles called proto-lysosomes after their scission, and these proto-lysosomes will then become functional after a maturation step [157,158]. The depletion of both spatacsin and spastizin leads to a reduced tubulation in HeLa cells, which prompted the proposition that these proteins are necessary for the initiation of the tubulation [38]. This involvement is consistent with the global observation of an accumulation of autophagosomes and/or lysosomal structures that can be accompanied by a depletion of free lysosomes in various models, including fibroblasts from SPG11 and SPG15 patients [38,39,42], brain sections of *Spg11*^−/−^ and *Spg15*^−/−^ mouse models [40] and MEFs of both *Spg11*^−/−^ and *Spg15*^−/−^ mouse models [40,41], even if some discrepancies can be noted between the different reports or models, including animal models. Moreover, spatacsin could have another role in a later step in ALR since the recruitment of dynamin at the lysosomal structures, involved in the proto-lysosome scission from the tubule [38], is decreased in *Spg11*^−/−^ mouse neurons [16].

At least two *Spg11*^−/−^ mouse models have been established, based on two different approaches [41,170]. An accumulation of autolysosomes in neurons of the *Spg11*^−/−^ mouse model has been reported in the former [41]. In the latter, neurons of *Spg11*^−/−^ mouse present enlarged lysosomes with an accumulation of gangliosides linked to an impaired lysosome membrane recycling, as seen in iPSC-derived human neurons from a cortical organoid model [16,170]. Interestingly, gangliosides accumulation has been linked to neurodegeneration, given that increased glutamate-induced neuronal death has been observed and that inhibition of gangliosides accumulation in lysosomes by treatment with miglustat, an inhibitor of glucosylceramide synthase, increases the neuronal survival and improves the motor phenotype induced by *Spg11* knock-down in zebrafish [16].

In addition to their roles in starvation-induced ALR, spatacsin and spastizin could also have a role in lysosome reformation in conditions of basal autophagy since an accumulation of autolysosomes has also been observed under feeding conditions in spatacsin- or spastizin-depleted cells [38]. This process seems to be especially important for neuronal cells, which have a reduced reaction to starvation and rely more on basal autophagy [171]. Therefore, loss-of-function mutations in *SPG11* and *ZFYVE26* would lead to global lysosomal renewal defects.

### 3.4. Crossroads between Endocytic and Autophagic Pathways

As previously mentioned, autophagy and endocytosis are two key, deeply interconnected, cellular processes. Crossroads between them can be seen at different levels of the pathways, notably the fusion between late endosomes and autophagosomes to form hybrid structures called amphisomes. Once formed, the amphisome can fuse with a lysosome to lead to the formation of an autolysosome to allow the degradation of the contained material [172] (Figure 1). It has been proposed that fusion between endosomes and autophagosomes is involved in the maturation of the autophagosome and that functional MVBs are needed for efficient autophagy [173]. A defect in this process can lead to an autophagic defect that can be linked to neurodegenerative diseases [5]. Despite spatacsin and spastizin interaction, an additional role for spastizin has been proposed. Indeed, spastizin is involved in the maturation of the autophagosomes since its depletion leads to an accumulation of immature autophagosomes in SPG15 patients’ cells [42]. This defect would be due to the incapacity of the mutated spastizin to interact with Rab5A and Rab11, leading to a more pronounced autophagy defect [42,43]. Rab5A localizes to early endosomes and Rab11 localizes to recycling endosomes and MVBs, and both proteins are necessary for endosome–autophagosome fusion at different levels [43,174,175].

Crossroads between endocytic and autophagic pathways involving HSP proteins have also been reported recently. Indeed, mutations in the *AP5Z1* gene, encoding for a subunit of the AP-5 complex, have also been identified as causative in SPG48 [164]. AP-5 stably interacts with spatacsin and spastizin and its expression is dependent on *SPG11* and *SPG15* gene expression but not reciprocally, therefore a participation of AP-5 in spatacsin and spastizin cellular functions is very likely [58,166]. Although a decrease in tubule formation from autolysosomes in MEFs of the *Ap5z1*^−/−^ mouse has been observed, as in SPG11 and SPG15 models, in conditions of starvation-induced autophagy [56,57], it has been suggested that the main role of AP-5 is in the endolysosomal system. Indeed, an accumulation of enlarged lysosomal structures identified as enlarged endolysosomes with abnormal storage material has been reported in SPG48 patient-derived fibroblasts [58]. In addition, recent investigations showed that AP-5 knockdown impairs retrograde trafficking of the cation-independent mannose 6-phosphate receptor (CIMPR) from endosomal compartments towards the TGN, which may indicate that AP-5 also acts on an alternative retrograde trafficking route [59]. Interestingly, axonal swellings with clustering of organelles and LAMP1-positive structures have been observed in neurons of the *Ap5z1*^−/−^ mouse model, suggesting its importance in neuronal cells [56].

Additionally, the protein VCP, involved in various neurodegenerative diseases, including HSP [73], is necessary for autophagosome maturation in basal autophagy and proteasome-inhibition conditions [76], in addition to its role in conjunction with strumpellin (SPG8) in endocytosis, as discussed above.

Finally, it has been suggested that Rab3GAP2, whose loss of function causes SPG69 [13,176], is involved in autophagy at different levels: autophagosome formation and autophagosome–autolysosome maturation [67,68]. Rab3GAP2 is the noncatalytic subunit of the heterodimeric Rab3GAP complex, which has been proposed to regulate Rab18 activity and autolysosome maturation. Indeed, autophagy defects are observed in a Rab3GAP2-deficient Drosophila model [68]. In contrast, another study has also suggested a role of Rab3GAP in autophagy but earlier in the pathway since it was shown that, in human primary fibroblasts, depletion in Rab3GAP2 led to an impaired lipidation of ATG8 family members necessary for autophagosome formation and the autophagy pathway [67]. Rab3GAP2 cellular functions are therefore still unclear but the available data in different models of diseases suggest that this protein could be involved in autophagy. Of note, mutations in *Rab3GAP2* have also been involved in the Martsolf syndrome and mutations in *Rab3GAP2*, *Rab3GAP1* and *Rab18* can give rise to the Warburg micro syndrome (clinically overlapping with the Martsolf syndrome and characterized by brain, eyes and endocrine abnormalities), further showing a link between these three proteins [68,177,178].

## 4. Lessons and Perspectives

Defects in the endolysosomal system and autophagy are a common pathological mechanism in several diseases and especially in neurodegenerative diseases, such as Alzheimer’s disease, Parkinson’s disease, Huntington’s disease and ALS, as strongly highlighted by numerous reviews [179,180,181,182,183,184,185]. For example, in the case of Parkinson’s disease, the endolysosomal pathway has been pinpointed as a key cellular pathway in the pathological mechanism of the disease and mutations in the gene encoding the glucocerebrosidase, a lysosomal enzyme, represent one of the most common genetic risk in Parkinson’s disease [183,186].

HSPs belong to motor neuron diseases which are a group of heterogeneous diseases in which overlaps at the clinical and genetic levels can be found. These pathways are particularly involved in motor neuron diseases, since they are altered not only in HSP, but also frequently in ALS and CMT disease, which are both motor neuron diseases [185,187,188]. For example, mutations of the genes encoding KIF5A, VCP and spatacsin are involved in subtypes of HSP but also in ALS and CMT disease [78,79,130,131,162]. In addition, other proteins of the pathways, not mutated in HSPs, have been founded mutated in motor neuron diseases like *CHMPB2* mutations in ALS [90,91].

The recurrence of the implication of these pathways in neurodegenerative diseases indicates that the nervous system is particularly sensitive to the disruption of the endolysosomal and autophagic systems. This can be explained by three main reasons. First, neurons, as post-mitotic cells, are not able to eject undesired material during cell division, which means that autophagy defects in these cells have particularly high impacts. Second, these pathways have additional specific functions in neuronal cells such as the maintenance of the synaptic process and membrane trafficking along the axon. Finally, the unique morphology of neurons exerts high demands on accurate and coordinated delivery of proteins and lipids to their final destination [117]. This point is critical in the pathophysiology of HSP where motor neurons are the most affected and their degeneration is responsible for the major clinical signs.

Because of the need for accurate regulation of those processes, any perturbation can give rise to a plethora of events. As we pointed out before, many HSP proteins impact the global membrane and protein trafficking in the cells. However, the nature of the alterations varies considerably. For example, they can be caused by a defect in receptor trafficking (e.g., BMP signaling), failure to deliver cargo protein to the correct destination (AP-4), the inability to degrade substrates by autophagy (TECPR2/SPG49), or impairment of lysosome formation (spatacsin/SPG11 and spastizin/SPG15). In neurons, these defects lead to similar pathological phenotypes of particular interest, such as the axonal swellings that have been observed in SPG4, SPG48 and SPG51 [55,56,189,190] or defects in neurite architecture that have been seen in SPG3A, SPG4, SPG6, SPG8, SPG11, SPG20 and SPG39 for instance [26,35,49,51,191,192,193]. A better comprehension of the involvement of the endocytic and autophagic pathways in the altered neuronal phenotype could help us understand the complexity of this continuum and therefore lead to the development of common therapeutics.

As of now, no curative treatment for any form of HSP is available on the market. A few therapeutics are used to relieve patients, mainly targeting a reduction of the spasticity. The mutations are often affecting not only one but multiple pathways and the interplay between the mitochondrion compartment, the microtubule cytoskeleton, the endolysosomal/autophagy pathway and the tubular endoplasmic reticulum seems critical in HSP pathogenesis [11]. Regarding the endolysosomal and autophagy systems, attempts to increase their efficiency have been tested in various models of neurodegeneration with variable success, particularly acting on mTOR and TFEB levels, but these did not include HSP [194,195]. Some drugs are currently in development for HSP forms related to these pathways and have been tested in vitro. For example, tideglusib, an inhibitor of GSK3 activity, has been tested on brain organoids derived from cells of SPG11 patients and show a rescue of the premature neurogenesis induced by SPG11 mutation [196]. In addition, drugs have also been tested on animal models of HSP, notably zebrafish. Miglustat, an inhibitor of the glucosylceramide synthase known to decrease the levels of GM2 gangliosides, has been tested on zebrafish with a knock-down of Spg11 and allows the partial rescue of the motor phenotype [16]. Additionally, modulation of BMP signaling is an interesting target. Dorsomorphin, a small molecule that blocks the kinase activity of BMP receptors, has been tested in zebrafish depleted in atlastin-1 and is able to rescue the motor phenotype [26]. However, these drugs have not yet reached clinical trials.

Thus, there is still an unmet need for therapeutics, and a clearer understanding of endolysosomal and autophagy pathways alterations may pinpoint new therapeutic targets. It is striking to note that in many HSPs at least one step of the endolysosomal and autophagic pathways is impaired. The consequences of these alterations have been observed in patients and models, indicating that the regulation of all the machinery involved is very sensitive to any modification. Finding therapeutics may then be a real challenge. The induction or inhibition of the whole or a part of the endolysosomal or autophagy systems may not be efficacious and may even be deleterious, and therefore targeting a selective marker would probably be more beneficial than non-specific modulation.

Finally, it still remains puzzling why various disease presentations can occur due to mutations in the same gene, and the autophagy/endolysosomal pathways are no exception. This is the case, for example, for mutations in *SPG11* accounting for diseases affecting the first (SPG11) or second (CMT) or both upper and lower motor neurons (ALS5). In the case of mutations in *KIF1A*, some phenotype–genotype correlations emerged recently. The diversity of *KIF1A* mutations, their inheritance models and associated phenotypes, while still puzzling for most mutations, seems due to dominant negative effects, haploinsufficiency or loss of function mechanisms according to the mutation and its location [197,198,199]. Understanding these mechanisms is critical for future personalized therapeutics and therefore multi-center reports of cohorts of patients mutated in these rare genes is still important [200].

## Data Availability

Not applicable.

## Abbreviations

ALR	Autophagic lysosome reformation
ALS	Amyotrophic lateral sclerosis
AP	Adaptor protein
ATG	Autophagy-related
BMP	Bone morphogenic protein
BMPRI	Type I BMP receptors
BMPRII	Type II BMP receptors
CAV1	Caveolin1
CEDNIK	Cerebral dysgenesis, neuropathy, ichthyosis, and keratoderma
CGN	*Cis*-Golgi network
CHMP	Charged multivesicular body proteins
CIMPR	Cation-independent mannose 6-phosphate receptor
CMT	Charcot–Marie–Tooth
EE	Early endosomes
EGFR	Epidermal growth factor receptors
ER	Endoplasmic reticulum
ESCRT	Endosomal sorting complexes required for transport
FTD	Frontotemporal dementia
GARP	Golgi-associated retrograde proteins
HOPS	Homotypic fusion and vacuole protein sorting
HSAN	Hereditary sensory and autonomic neuropathy
HSP	Hereditary spastic paraplegia
IBMPFD	Inclusion body myopathy with Paget disease and frontotemporal dementia
ILV	Intraluminal vesicles
LC3	Light chain 3
LIR	LC3-interacting region
M6PR	Mannose-6-phosphate receptor
MEF	Mouse embryonic fibroblast
MIT	Microtubule interacting and trafficking
MVB	Multivesicular body
PAS	Phagophore assembly site
SNAP29	Synaptosomal-associated protein 29
SNARE	Soluble N-ethylmaleimide-sensitive factor attachment protein receptors
SPG	*Spastic Paraplegia Gene*
SPICT	Spichthyin
STX17	Syntaxin 17
TECPR2	Tectonin beta-propeller repeat containing 2
TGFβ	Transforming growth factor beta
TGN	*Trans*-Golgi network
VAMP8	Vesicle-associated membrane protein 8
VCP	Valosin-containing protein
WASH	Wiskott–Aldrich syndrome protein and SCAR homolog
